# TCR Convergence in Individuals Treated With Immune Checkpoint Inhibition for Cancer

**DOI:** 10.3389/fimmu.2019.02985

**Published:** 2020-01-09

**Authors:** Timothy John Looney, Denise Topacio-Hall, Geoffrey Lowman, Jeffrey Conroy, Carl Morrison, David Oh, Lawrence Fong, Li Zhang

**Affiliations:** ^1^Thermo Fisher Scientific, South San Francisco, CA, United States; ^2^OmniSeq Inc., Buffalo, NY, United States; ^3^Roswell Park Comprehensive Cancer Center, Buffalo, NY, United States; ^4^Division of Hematology and Oncology, Helen Diller Family Comprehensive Cancer Center, University of California, San Francisco, San Francisco, CA, United States

**Keywords:** biomarker, immune repertoire analysis, T cell repertoire, checkpoint blockade immunotherapy, convergence, AmpliSeq™, antigen stimulation, Ion Torrent next-generation sequencing

## Abstract

Tumor antigen-driven selection may expand T cells having T cell receptors (TCRs) of shared antigen specificity but different amino acid or nucleotide sequence in a process known as TCR convergence. Substitution sequencing errors introduced by TCRβ (TCRB) repertoire sequencing may create artifacts resembling TCR convergence. Given the anticipated differences in substitution error rates across different next-generation sequencing platforms, the choice of platform could be consequential. To test this, we performed TCRB sequencing on the same peripheral blood mononuclear cells (PBMC) from individuals with cancer receiving anti-CTLA-4 or anti-PD-1 using an Illumina-based approach (Sequenta) and an Ion Torrent-based approach (Oncomine TCRB-LR). While both approaches found similar TCR diversity, clonality, and clonal overlap, we found that Illumina-based sequencing resulted in higher TCR convergence than with the Ion Torrent approach. To build upon this initial observation we conducted a systematic comparison of Illumina-based TCRB sequencing assays, including those employing molecular barcodes, with the Oncomine assay, revealing differences in the frequency of convergent events, purportedly artifactual rearrangements, and sensitivity of detection. Finally, we applied the Ion Torrent-based approach to evaluate clonality and convergence in a cohort of individuals receiving anti-CTLA-4 blockade for cancer. We found that clonality and convergence independently predicted response and could be combined to improve the accuracy of a logistic regression classifier. These results demonstrate the importance of the sequencing platform in assessing TCRB convergence.

## Introduction

Checkpoint blockade immunotherapy (CPI) may elicit durable anti-tumor responses in a subset of individuals with cancer. Identifying predictive biomarkers to guide treatment selection remains a primary goal of immune-oncology translational research. Owing to limitations in the quantity and quality of available tumor material, and its use in routine PD-L1 immunohistochemistry testing, there is a pressing need to identify non-invasive biomarkers derived from peripheral blood. Within this context tumor mutation burden (TMB) has drawn attention as a potential predictive biomarker for response to CPI, under the premise that it may serve as a surrogate for total neoantigen load and thus the sensitivity of a tumor to immunotherapy. Originally measured from tumor biopsy material, TMB measurements have now been demonstrated from next generation sequencing of peripheral blood cfDNA (1). Unfortunately, accumulating evidence suggests the predictive value of this biomarker may be limited (2), with recent analyses of TMB in mono- or combination CPI for non-small cell lung cancer (NSCLC) indicating an overall area under the receiver operator characteristic (ROC) curve (AUC) of 0.60 and 0.68, respectively, for predicting durable clinical benefit (3, 4), comparable to the accuracy of PD-L1 IHC, while results of Checkmate 026, a study of first-line Nivolumab for NSCLC, revealed no difference in overall survival in subjects stratified by TMB (5). Importantly, TMB is unable to identify immunogenic, CPI sensitive tumors having neoantigens other than those derived from non-synonymous mutations, as has been demonstrated by studies of TMB in polyoma virus-associated Merkel cell carcinoma and renal cell carcinomas (6).

Motivated by the shortcomings of existing non-invasive biomarkers, here we evaluated the use of peripheral blood TCRB repertoire sequencing as a source of predictive biomarkers for response to CTLA-4 monotherapy for cancer. Previous TCR sequencing studies have evaluated T cell clonal expansion as a stand-alone predictive biomarker, with mixed results (7, 8). One outstanding question is whether TCR sequencing may be used for *in silico* identification of tumor antigen specific T cells, given that the frequency of such cells could serve as a direct measurement of tumor immunogenicity. Although there are no known methods to predict the antigen specificity of a TCR from nucleotide sequence, we hypothesized that the central role of chronic antigen stimulation in the emergence of cancer would provide a means to infer the presence of tumor antigen specific T cells, given that sustained antigen-driven selection may give rise to convergent T cell receptors having a shared antigen specificity (i.e., identical amino acid sequence) but different nucleotide sequences. Unlike biomarkers relying of the quantification of tumor genetic alterations, TCR convergence: (1) may detect T cell responses to tumor neoantigens beyond those arising from non-synonymous mutations; (2) avoids probabilistic models for prediction of immunogenicity; (3) is sequencing efficient, typically requiring <2 M reads per sample; and (4) may be measured from the abundant genetic material within the buffy coat fraction of centrifuged peripheral blood to enable liquid biopsy applications.

Despite these advantages, efforts to evaluate TCR convergence may be hampered by the sensitivity of this feature to substitution sequencing errors, which may create artifacts resembling convergent TCRs. To circumvent this issue, here we leveraged the low substitution error rate of the Ion Torrent platform to evaluate convergence as a predictive biomarker for response to anti-CTLA-4 monotherapy in a set of 22 study subjects with cancer. For context, we compared convergence values obtained using this platform to those for the same samples interrogated with Illumina-based TCRB repertoire sequencing. Finally, we examined whether TCR convergence may be combined with measurements of clonal expansion to improve prediction of immunotherapy response.

## Materials and Methods

### Peripheral Blood Samples

Eight peripheral blood leukocyte (PBL) samples were obtained from longitudinal blood draws from three anti PD-1 treated melanoma study subjects (donor 1: three samples; donor 2: three samples; donor 3: two samples) at the University of California San Francisco (UCSF). The average time between consecutive blood draws was 4 weeks. Baseline (pre-treatment) PBL were collected from 22 cancer study subjects treated with CTLA-4 monotherapy (Ipilimumab) at Roswell Park Cancer Research Institute or UCSF. Samples were collected within 1 week of administration of the first CTLA-4 dose. Response was evaluated using RECIST criteria.

### TCR Sequencing

For the Ion Torrent-based approach, RNA was extracted from cryopreserved buffy coat straws using the Qiagen RNeasy Midi Kit (Qiagen Cat. No. 75144). Extractions were performed over multiple days at two different sites. Purified RNA samples were quantified using Qubit RNA HS Assay Kit (Thermo Fisher Scientific Cat. No. Q32852). The Agilent 2100 Bioanalyzer and Agilent RNA 6000 Nano Kit were used to quantify and evaluate RNA integrity. Twenty-five nanogram of total RNA was reverse transcribed using SuperScript IV VILO Master Mix (Thermo Fisher Scientific Cat. No. 11756050). For each sample, 25 ng cDNA was amplified using the Oncomine TCR Beta-LR Assay (Thermo Fisher Scientific Cat. No. A35386), and protocol as described in the Oncomine TCR Beta Assay User Guide MAN0017438 Revision A.0. Libraries were purified with Agencourt AMPure XP beads (Beckman Coulter Cat. No. A63880), washed with 70% ethanol, and eluted in 50 μL Low TE buffer. Resulting library samples were diluted 1:100 and quantified using the Ion Library Quantitation Kit (Thermo Fisher Scientific Cat. No. 4468802), then diluted to 25 pM with Low TE buffer. Equal volumes from 8 samples at a time were pooled together for sequencing on one Ion 530 chip, followed by analysis via Ion Reporter version 5.10. TCR sequencing with the Illumina-based platform was performed by Sequenta as described in Klinger et al. (9), the ImmunoSeq assay (Adaptive Biotechnologies) or Archer Immunoverse HS TCRB assay, per manufacturer instruction.

### Sequencing of 30 Reference TCRB Rearrangements

Thirty TCRB rearrangements presented in Sandberg et al. (10) were cloned into plasmids via GeneArt. Plasmids were pooled at 0.1 fg each prior to sequencing via the Oncomine and ImmunoSeq assays at either low (survey) or high (deep) depth. To minimize differences owing to library preparation, the same PCR replicate structure was used for both assays. For survey and deep level, two or six Oncomine libraries were separately prepared and sequenced to ~1 M reads depth, then bioinformatically combined for analysis, consistent with the PCR replicate structure employed by the ImmunoSeq assay. The ImmunoSeq assay was run per manufacturer instruction by a contract research organization.

### Sorting and TCR Sequencing of Peripheral Blood T Cells

1E3, 1E4, or 1E5 CD3+ T cells from a healthy donor were sorted into CTS™ OpTmizer™ (Thermo Fisher Scientific Cat. No. A1048501) serum free cell culture media and stimulated with CTS™ anti-CD3/CD28 Dynabeads (Thermo Fisher Scientific Cat. No. 40203D) for 4 days prior to extraction of total RNA. The entirety of the extracted RNA was used for library preparation, followed by sequencing to saturation, as determined by downsampling analysis via Ion Reporter, version 5.12.

### Calculation of TCR Convergence and Clonality

TCR convergence was calculated as the aggregate frequency of clones (here defined as unique TCRB nucleotide sequences) sharing a variable gene (excluding allele information) and CDR3AA sequence with at least one other identified clone. For the Oncomine TCRB-LR assay, TCR convergence is pre-calculated from the set of identified clones and provided as a standard output. TCR convergence values in Illumina-based sequencing data were calculated with a custom R script [https://github.com/mlizhangx/TCR-3D] in an identical manner to that for the TCRB-LR assay. Shannon diversity was calculated using the set of clone frequencies (*p*) as indicated below:

$$\begin{array}{l} -\overset{N}{\underset{i=1}{\sum }}p_{i}\log_{2}\left(p_{i}\right) \end{array}$$

while the normalized Shannon entropy (i.e., evenness) was calculated by dividing the Shannon diversity by log_2_(*N*), where *N* is the total number of detected clones. Clonality was defined as 1 − normalized Shannon entropy (11).

### Comparison of Repertoire Features Across Sequenta and Oncomine TCRB-LR Datasets

The proportion of overlapping clones between two timepoints within the same subject was evaluated by Jaccard index. Spearman correlation coefficient (“cor.test” in R “stats” library) was used to compare each repertoire feature across Sequenta and Oncomine TCRB-LR Datasets. Statistical significance was declared based on *p* < 0.05. No multiple testing adjustment was carried out.

### Analysis of Clonality and Convergence Values in ImmunoSeq Data

TCR convergence and clonality values were calculated by analysis of publicly available clonotype files derived from Emerson et al. (12) (*N* = 666) using the information in the “amino_acid” and “v_gene” columns and a method identical to that applied to the Sequenta and Oncomine TCRB-LR datasets. In instances where the “v_gene” value was reported as “unresolved,” the v_gene was assigned to the value in the “v_family” column. The analysis excluded rearrangements having a “productive_frequency” column value of “null.”

### Modeling TCR Convergence Values in ImmunoSeq Data

We attempted to model the likelihood that a given clone would be identified as a member of a convergent group. The model took into account: (1) the CDR3 length of each clone, obtained by taking the length of sequence in the “amino_acid” column of the clonotype table and eliminating anchor residues; (2) the probability that a random single base CDR3NT substitution within a productive rearrangement would be synonymous, estimated to be ~1 in 4 based on the codon table; (3) the number of reads per clone, taken from the “reads” column of the clonotype table; and (4) the substitution sequencing error rate per base. This is a simple model that does not take into account motif-specific error hotspots, codon usage biases within the CDR3, nor does it account for residual PCR-derived substitution errors. Hence it should be considered a rough approximation to be used for exploratory purposes. We tested the model using substitution error rates ranging from ~1E-2–1E-5 errors per base, using estimates from literature as a starting point. A Spearman correlation was used to assess model fit. Python code used for model exploration is found on (https://github.com/mlizhangx).

### Sensitivity Analysis of Oncomine TCRB and Archer Immunoverse HS TCR Beta Libraries

Total RNA from ~5 M PBMCs was divided into two pools and spiked with Jurkat total RNA at 1:10E5 or 1:10E6 mass ratio. The RNA pools were then used for library preparation via the Oncomine TCRB-LR Assay, the Oncomine TCRB-SR assay (utilizing framework 3 and joining gene primers and a similar AmpliSeq-based library preparation method), or the Archer Immunoverse HS TCR beta assay, using from 25 to 300 ng RNA as input for library preparation. Two libraries were prepared for each input condition and each assay. This procedure was then repeated using PBMC RNA from a second donor. Oncomine TCRB-LR assay libraries were sequenced to a target of 2 M reads depth using the S5 530 chip, while the Oncomine TCRB-SR assay libraries were sequenced to >20 M reads each using the S5 550 chip. Archer Immunoverse HS TCR beta libraries were sequenced to a target of >20 M reads each using the Illumina MiSeq. For comparison between Archer Immunoverse HS TCR beta and Oncomine TCRB-LR assays, Immunoverse HS TCR beta data was downsampled to an equivalent read depth (~2 M reads per library) prior to analysis via the Immunoverse HS TCR beta software (Archer Analysis). For comparisons between the Oncomine TCRB-SR and the Archer Immunoverse HS TCR beta, the entirety of the reads was used for analysis. TCR convergence values were obtained directly from the Ion Reporter analyzer for the Oncomine assays, or, for the Archer Immunoverse HS TCR beta assay, calculated using a custom script in an identical manner. All library preparations and analyses were performed independently by a contract research organization.

### Logistic Regression-Based Prediction of Response and Model Scoring

A logistic regression model with the clinical response status as the binary outcome and TCR convergence and clonality as the predictors was trained using the function “train” from the R “caret” library with the following parameters: method = “glm” and family = “binomial.” Model scores represent the response probability values obtained by applying the caret function “predict,” with the trained model and the sample clonality and convergence values as inputs. Model performance was evaluated using the function “roc” from the R “pROC” library. Area under the receiver operator characteristic (ROC) curve (AUC) was calculated using the function “auc” from the same R library. The same functions were used to evaluate the performance of TCR convergence and clonality as stand-alone biomarkers. Cross-validation was performed by training the logistic regression classifier using the trainControl feature of the caret package with the parameters: method = “lgocv,” number = 2,000, *p* = 0.75, classProbs = True, and savePredictions = “final.” Models were separately trained using both TCR convergence and clonality as the predictors, or either feature alone. The optimal score threshold for distinguishing responders from non-responders, and the sensitivity, specificity, and positive predictive value at the optimal threshold, were obtained using the “coords” function from the pROC library with best. method = “youdens” and ret = c(“threshold,” “specificity,” “sensitivity,” “ppv”).

## Results

### Cross-Platform Analysis of Repertoire Features

**Figure 1:**
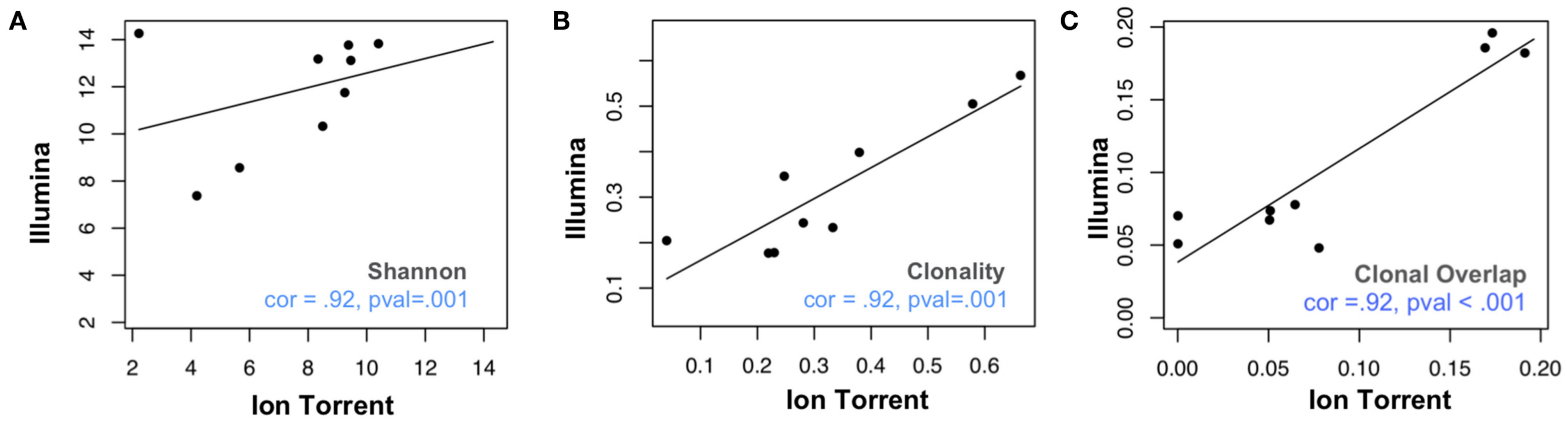
Comparative analysis of repertoire features in samples analyzed via Ion Torrent and Illumina-based assays. Eight peripheral blood leukocyte (PBL) samples derived from three donors were analyzed using the Oncomine TCRB-LR assay (Ion Torrent, X-axis) or Sequenta TCRB assay (Illumina, Y-axis). Pearson's correlation coefficient was used to measure the consistency of two platforms with respect to **(A)** clone diversity (Shannon entropy), **(B)** clonality (normalized Shannon entropy), and **(C)** clonal overlap. Blue dashes indicate position of identity line.

We assessed 8 samples from anti-PD1 treated melanoma subjects using a commercial Illumina-based TCRB sequencing assay (Sequenta; now part of Adaptive Biotechnologies; service discontinued) and the Ion Torrent based Oncomine TCRB-LR assay. For each sequenced library, we evaluated the number of clones detected, Shannon diversity index, clonality (i.e., 1–normalized Shannon entropy) and the frequency of convergent TCRs (section Materials and Methods). Within each subject from the same sequencing platform, clonal overlap between the samples from any two time points for the same subject was calculated by Jaccard index. We found measurements of Shannon diversity index, clonality, and clonal overlap to be significantly correlated between the two platforms (Figures 1A–C, Pearson's correlation = 0.88; *p* = 0.007). We also note a trend toward higher clonality values in the Oncomine dataset (*p* = 0.04, Wilcoxon signed sum test, Supplemental Figure 1A), potentially due to the differences in the number of clones detected across the assays.

### Assessment of TCR Convergence

**Figure 2:**
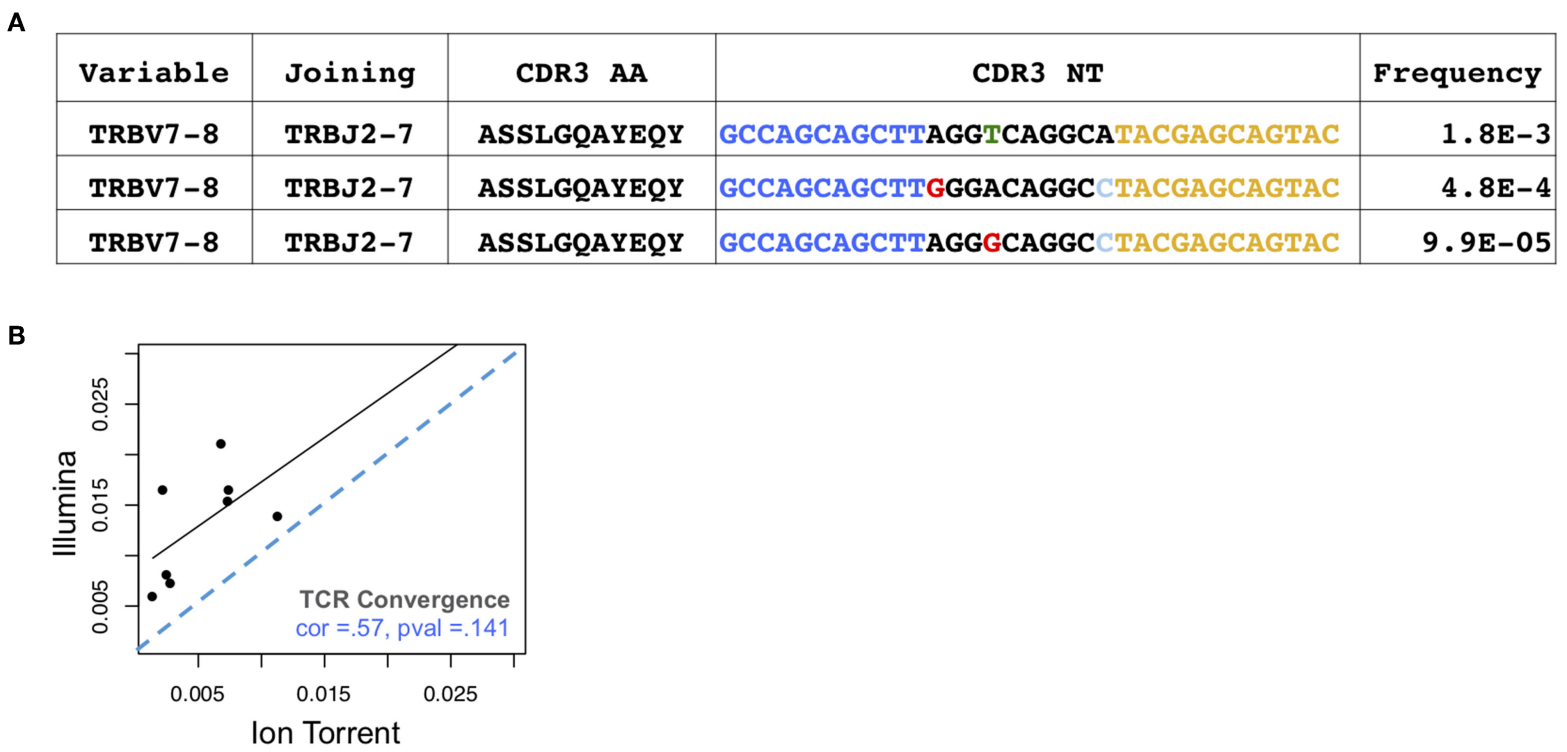
Assessment of TCR convergence. **(A)** Example of a convergent TCR group detected in the peripheral blood of an individual with melanoma. This group consists of three TCRβ clones that are identical in TCRβ amino acid space but have distinct CDR3 NT junctions owing to differences in non-templated bases at the V-D-J junction. Blue indicates bases contributed by the variable gene while yellow indicates bases contributed by the joining gene. Red arrows indicate positions where clones differ. Substitution sequencing errors and PCR errors can create artifacts that resemble convergent TCRs. **(B)** TCR convergence, calculated as the aggregate frequency of clones sharing an amino acid sequence with at least one other clone. Blue dashes indicate position of identity line.

Antigen-driven responses should result in the expansion of multiple T cell clones that recognize a given antigen. TCR convergence is defined as the aggregate frequency of clones sharing a variable gene (excluding allele information) and CDR3AA sequence with at least one other identified clone. An example of a convergent TCR group identified in an individual with melanoma is presented in Figure 2A. For the Oncomine TCRB-LR assay, TCR convergence is pre-calculated from the set of clones reported by the Ion Reporter software and provided as a standard output. We hypothesized that the choice of sequencing platform would be consequential for the measurement of TCR convergence given that substitution sequencing errors may mimic TCR repertoire diversity deriving from N-additions and exonucleotide chewback within the V-D and D-J junctions of the CDR3. We found that convergence measurements were not significantly correlated (Figure 2B, Spearman correlation = 0.33; *p* = 0.43), with eight of eight Illumina-based Sequenta libraries showing higher TCR convergence compared to the corresponding Ion Torrent libraries (*p* = 0.002, Wilcoxon signed rank test, Supplemental Table 1, Sheet 2).

### Assessment of TCR Convergence in ImmunoSeq Data

The elevated convergence values observed in the Sequenta data could reflect aspects of the Sequenta library preparation and analysis protocol, rather than platform-specific sequencing errors. To assess the generalizability of this result we next evaluated convergence values in a large public dataset (12) (*N* = 666) produced by the Illumina-based ImmunoSeq assay, which employs a distinct library preparation and analysis protocol. Strikingly, while the clonality values for these samples were within the range of those observed in Oncomine or Sequenta derived libraries (Supplemental Figure 1A), the convergence values were significantly higher (Supplemental Figure 1B). To better understand this result we assessed the relationship between the clonality and convergence values of each ImmunoSeq sample. Surprisingly, TCR clonality and convergence were highly correlated (Spearman cor = 0.89, *p* < 2E-26, Supplemental Figure 1C) in the ImmunoSeq dataset, but far less so in Oncomine data (Spearman cor = −0.33, *p* = 0.09, Supplemental Figure 1D). Hypothetically, the strong correlation between TCR convergence and clonality could reflect the fact that samples having higher clonality tend to have higher frequency clones that are sequenced many times. In the context of a non-negligible substitution sequencing error rate, such highly sequenced clones may spawn artifacts that lead to false convergent events. To test this possibility we asked whether the observed ImmunoSeq convergence values could be recapitulated by a model taking into account the sequencing depth per clone, and the likelihood that a substitution error would give rise to a synonymous mutation within the CDR3 of each clone (section Materials and Methods). We tested the model over a range of potentially plausible residual substitution error rates. We found that the model closely fit the observed convergence values (Spearman cor = 0.85 at a residual substitution error rate of 8.5E-3 errors per base, Supplemental Figure 2, black dots), supporting the potential existence of substitution sequencing errors in this dataset.

### Cross-Platform Analysis Using Reference Rearrangements

The above finding provided indirect evidence that TCR convergence values are elevated in ImmunoSeq data compared to the Oncomine and Sequenta data, potentially due to artifacts arising from PCR or sequencing errors. To directly address this question we sought to compare the two assays using the same sample. To aid in the interpretation of results, we created a set of reference plasmids representing the TCRB sequences from 30 common T cell lines that were used to validate the BIOMED-2 TCRB primer set (10). Plasmids were pooled at 0.1 fg per plasmid followed by library preparation and sequencing via the Oncomine or ImmunoSeq assays. Sequencing was performed at both survey and deep sequencing depths (section Materials and Methods). In this experiment, we expect each assay to report 30 rearrangements, while unresolved sequencing or PCR derived artifacts will lead to the reporting of more than 30 rearrangements. While both assays identified all 30 rearrangements, the ImmunoSeq assay reported a greater number of productive rearrangements than the Oncomine assay at both the low (54 vs. 30) and high (84 vs. 31) sequencing depths (Supplemental Table 2).

### Analysis of Sorted, Counted T Cells

Sequencing of rearrangements cloned into plasmids suggested that the Oncomine assay may report few artifactual rearrangements. However, it is unclear the extent to which this result may reflect performance of the assay with samples containing diverse T cell populations. To address this question we prepared and sequenced Oncomine libraries derived from 10E3, 10E4, and 10E5 sorted CD3+ peripheral blood T cells from a healthy donor having a polyclonal T cell repertoire, using the entirety of the RNA for library preparation, and sequencing the libraries to exhaustion (section Materials and Methods). In this experiment, although the sequence of each rearrangement is not known, the maximum number of unique rearrangements is bounded by the number of sorted T cells. We found that the number of clones detected closely matched expectation, consistent with the Oncomine assay having a high sensitivity but also a low frequency of artifacts (Supplemental Figure 3).

### Comparison to an Illumina-Based Assay Employing Molecular Barcodes

One question is whether molecular barcode based methods may mitigate the effect of PCR or sequencing derived errors. Molecular barcode-based methods have been shown to improve the detection of single nucleotide variants (13), but potentially at the expense of lower template molecule capture efficiency compared to standard PCR. For example, recent evaluations of molecular barcode-based TCRB repertoire sequencing protocols indicated a capture efficiency of ~1–3 TCRB cDNA transcripts per T cell (14, 15), compared to ~10 transcripts per cell for a nanoliter dPCR-based approach (15) having a capture efficiency comparable to conventional microliter-volume PCR (16). To further explore this issue, we compared the Oncomine TCRB assay to the Archer Immunoverse HS TCR beta assay, a molecular barcode based, RNA-compatible assay for the Illumina platform. Total RNA from a peripheral blood donor was divided into two pools, spiked with Jurkat RNA separately at 10E-5 and 10E-6 frequency, then used to prepare libraries via the Oncomine TCRB-LR assay, the Oncomine TCRB-SR assay (FFPE-compatible; see section Materials and Methods), or the Archer Immunoverse HS TCR beta assay. Compared to the Oncomine assays, the Archer Immunoverse HS TCR beta assay identified fewer clones and showed lower sensitivity than the Oncomine assays over a range of input amounts (Supplemental Tables 3A,B,D,E). Specifically, the assay was unable to detect the Jurkat spike-in clone at 10E-5 and 10E-6 from 300 ng input, while the Oncomine assays identified the clone in all replicates at this input level and could detect the Jurkat clone at 10E-5 in all 25 ng libraries. By contrast, convergent TCR frequency values were not significantly different across the two assays (*p* = 0.29 and *p* = 0.13, for Oncomine TCRB-LR vs. Immunoverse HS TCR beta and Oncomine TCRB-SR vs. Immunoverse HS TCR beta, respectively, by Wilcoxon signed rank test; Supplemental Tables 3C,F).

### Association With Response to CTLA-4 Monotherapy

**Figure 3:**
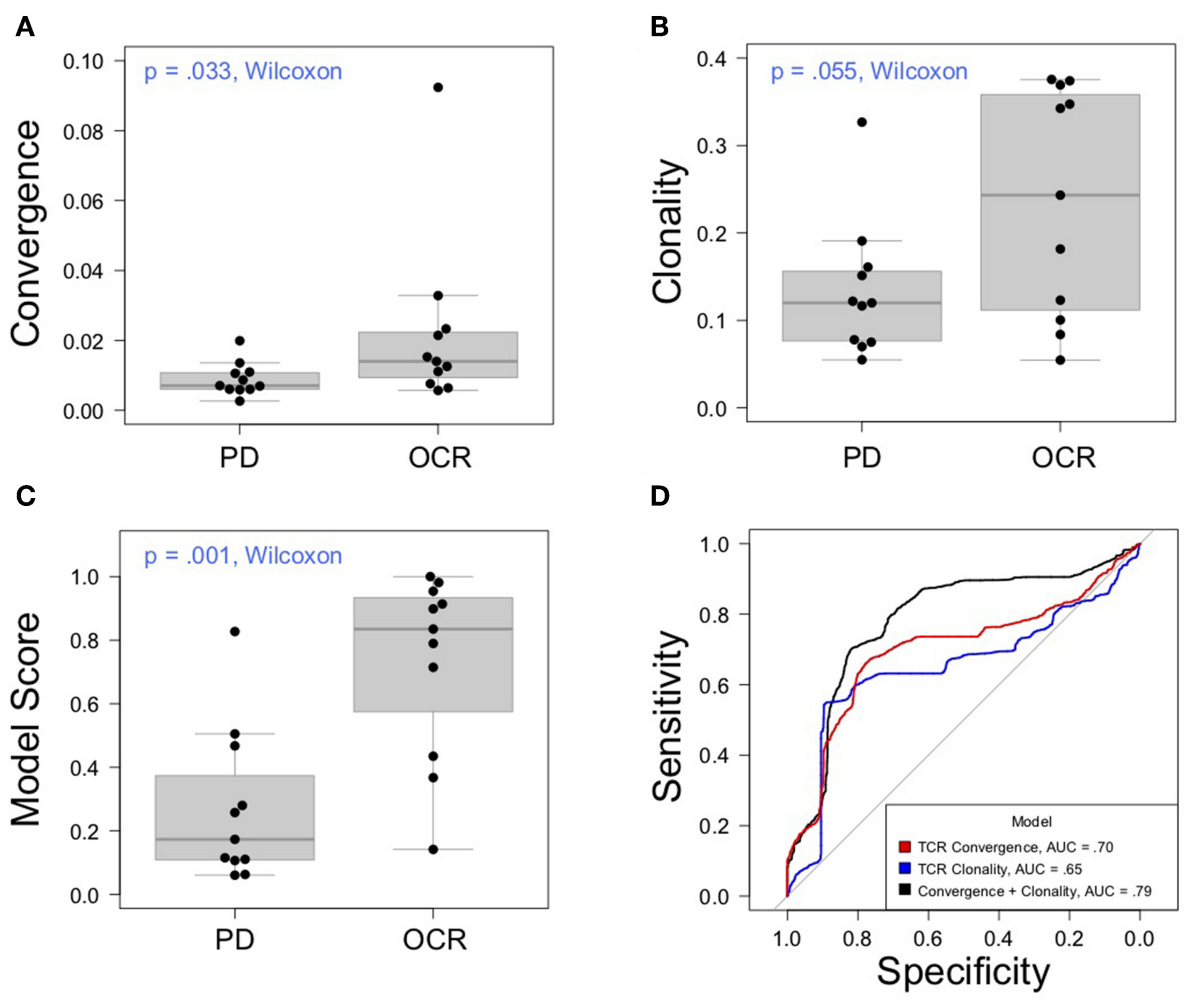
Association between clinical outcomes and TCR convergence. **(A)** TCR convergence and **(B)** clonality for responders (*N* = 11) and non-responders (*N* = 11) to CTLA-4 blockade for cancer. TCR clonality is calculated as 1—the normalized Shannon entropy of clone frequencies. Convergent TCR frequency was calculated as described in methods. All cancer types were included in the analysis. **(C)** Response probability scores from a logistic regression classifier trained using TCR clonality and convergence as features to predict response to immunotherapy. Score indicates likelihood that a sample is a responder. **(D)** Receiver operator characteristic curves derived from leave-group-out cross validation analysis of models using clonality, convergence, or the combination of clonality and convergence to predict immunotherapy response. ROC curves represent the average model performance following 2,000 random train-test splits, where 75% of the dataset was used to train the model followed by testing on the remaining 25%. The combination of TCR clonality and convergence shows better performance (AUC = 0.89) than models using TCR convergence and clonality alone (AUC of 0.70 and 0.65, respectively).

Having thoroughly evaluated the technical aspects of the Oncomine TCRB-LR assay, we next applied the assay to evaluate TCR convergence as a predictive biomarker for response to CTLA-4 blockade in a cohort of 22 individuals with RECIST graded response annotations (11 responders, 11 non-responders) representing three major cancer types: clear cell adenocarcinoma (two responders, zero non-responder), melanoma (seven responders, six non-responders) and prostate cancer (two responders, four non-responders). Response was defined as stable disease, partial response, or complete response following immunotherapy. Cancer annotations and repertoire features for this cohort are presented in Table 1, while detailed repertoire metrics for all samples are presented in Supplemental Table 1, Sheet 1. We found TCR convergence to be elevated in those who had an objective response to immunotherapy (*p* = 0.033, Wilcoxon sum rank test, Figure 3A) and could discriminate responders from non-responders with an AUC of 0.77. Given previous reports on the potential biomarker value of T cell clonal expansion, we next asked whether TCR clonality values differed between responders and non-responders. We observed a trend toward higher clonality in those who responded to immunotherapy (*p* = 0.055, Wilcoxon sum rank test, Figure 3B; AUC = 0.74). Given that clonal expansion and TCR convergence measure independent repertoire features, we trained a logistic regression classifier using TCR convergence and clonality as the two model features to test whether they might be combined to improve the prediction of response (section Materials and Methods). We found that the combination of convergence and clonality improved the prediction of response (Wilcoxon sum rank test *p* = 0.001, model response probability score for responders vs. non-responders; Figure 3C, AUC = 0.89). Finally, to evaluate model robustness, we performed repeated leave-group-out cross validation of the two feature models, and compared performance to the models using TCR convergence or clonality as a sole predictor of response. We found the two-feature model to outperform those using a single feature, achieving a specificity of 0.82, sensitivity of 0.71, and positive predictive value of 0.80 at the optimal threshold score, as determined by Youden's J (Figure 3D and section Materials and Methods).

**Table 1:** Cancer type and summary repertoire features for 22 individuals receiving CTLA-4 monotherapy.

**Category**	**Subdefinition**	**Responder**	**Non-responder**
Cancer type	Prostate	2	4
	Melanoma	7	6
	Adenocarcinoma	2	0
	Not indicated	0	1
	Total	11	11
Repertoire features	Clones detected	32,916 (5,168–56,231)	30,015 (5,894–58,222)
	TCR convergence	0.022 (0.006–0.092)	0.008 (0.002–0.019)
	Clonality	0.24 (0.055–0.376)	0.133 (0.055–0.327)

*Summary repertoire features and sample annotations for cohort. Each individual was profiled via the Oncomine TCRB-LR Assay at a single baseline timepoint using 25 ng of cDNA derived from PBL total RNA. Repertoire feature values indicate the average and range for responders and non-responders*.

## Discussion

The majority of TCRB sequencing data published to date has been generated using the Illumina platform, which has inherent substitution sequencing errors (17). Platforms having a lower substitution error rate could produce more suitable data. In this study, we compared TCRB repertoire data produced by the Ion Torrent Oncomine TCRB-LR and SR assays to data produced by three Illumina-based assays. Previous studies have evaluated the effect of platform specific sequencing errors on immune repertoire data (18), but to our knowledge the field has yet to examine the relevance of such errors to immunotherapy biomarker discovery. We hypothesized that the low substitution error rate of Ion Torrent platform might be critical for the measurement of convergence (19, 20). Indeed, in a side-by-side comparison of samples analyzed using Illumina-based Sequenta and Ion Torrent based Oncomine TCRB sequencing, we found measurements of TCR clonality, diversity and clonal overlap to be consistent across platforms, while TCR convergence values were not significantly correlated. Furthermore, a subsequent analysis of published ImmunoSeq data revealed elevated TCR convergence values that were highly correlated with sample clonality and could be accurately modeled as a suggested artifact derived from residual substitution sequencing errors. In reviewing literature, we note additional reports of TCR convergence in Illumina-based data that are significantly higher than those we observe in Ion Torrent data (21). Taken together, these results suggest the choice of sequencing platform may be consequential for biomarker applications of TCR convergence, and raise the possibility that TCR convergence may have been overlooked as a predictive biomarker owing to obfuscating platform specific noise. Beyond platform specific sequencing errors, other factors that may influence data quality include the number of PCR cycles used during library amplification, the fidelity of the chosen polymerase, but also the success of downstream informatics methods in eliminating PCR or sequencing errors. Indeed, among the three Illumina-based assays tested, we observed differences in the frequency of convergent TCRs, suggesting that assay protocol may significantly impact results.

Our results also highlight potential tradeoffs in the application of molecular barcodes to immune repertoire sequencing. In a comparison of the Oncomine TCRB assay with the molecular barcode based Archer Immunoverse HS TCR beta assay, we found that the latter assay detected fewer rearrangements and had a lower sensitivity than the Oncomine assay. However, this assay also reported convergence frequencies that were not significantly different than those reported by the Oncomine assay, suggesting that the use of molecular barcodes may reduce noise deriving from sequencing or PCR errors at the expense of lower sensitivity compared to conventional PCR based approaches.

Here we report elevated TCR convergence in baseline peripheral blood of those who respond to CTLA-4 blockade for cancer. We define TCR convergence as the aggregate frequency of T cell clones within an individual that share a variable gene and CDR3AA sequence with at least one other clone, but differ at the nucleotide level. This definition can be contrasted with instances in literature where this term has been used to refer to TCRB or IGH amino acid sequences found in more than one individual (22–24) (i.e., “public” rearrangements) or instances where researchers attempt to identify functionally equivalent TCRs that differ in amino acid space (25). Compared to the latter approach, we have adopted a stringent definition of convergence, with the goal of minimizing the false positive rate for the detection of convergent TCRs. Our approach builds upon the notion that false positive convergent events, either owing to the grouping of functionally dissimilar clones, or the presence of artifactual clones deriving from residual substitution errors, have the potential to conceal meaningful signal in TCR repertoire data, a possibility exacerbated by the rarity of bona fide convergence events. Consequently, the frequency of convergent TCRs reported here may underestimate the frequency of functionally equivalent T cell clones. Nonetheless, we note that functional or binding data would ultimately be required to prove shared tumor antigen specificity of convergent TCRs.

One proposed advantage of TCR convergence as a biomarker is its ability to detect T cell responses to tumor neoantigens beyond those arising from non-synonymous mutations. Although this dataset is small, we find that convergence values could discriminate responders from non-responders with significant accuracy (AUC = 0.77), comparing favorably to the historical performance of tumor mutation burden as a biomarker. The extent to which elevated TCR convergence is a feature of CPI sensitive tumors of other cancer types will be clarified by ongoing studies involving larger cohorts.

We hypothesize that T cells having convergent TCRs are likely to target tumor-associated antigens in those with cancer. However, these data do not shed light on the phenotype of such tumor antigen specific T cells. Given that chronic antigen stimulation may give rise to exhausted or dysregulated T cells having distinct expression levels of cell surface receptors, including inhibitory receptors (26), it is possible that FACS-based methods may be used to enrich for T cells having convergent TCRs. This possibility may be relevant given recent reporting of a phenotypically abnormal PD-1 high intratumoral T cell population in NSCLC subjects treated with PD-1 blockade, the frequency of which was found to be predictive of immunotherapy response (27).

One question arising from this work is whether additional insight would be gained by analysis of the unseen TCRα (TCRA) chains of T cells having convergent TCRB chains. Accepting that members of a convergent TCR group share antigen specificity, one can infer that either: (1) the unseen TCRA chains are functionally identical and help determine the antigen specificity of the receptor; or (2) convergent TCRs are TCRB chain dominant, while the TCRA chains play an accessory or stabilizing role but do not affect the antigen specificity of the receptor. Given that the unsequenced TCRA chains of group members are likely to differ in sequence space owing to the random nature of VDJ recombination, the latter case may be more likely. In this scenario, the full length TCRB chains of convergent TCRs could be paired with generic TCRA chains to efficiently generate tumor antigen specific TCRs. However, the proposition that beta chain dominance is a feature of TCRs having convergent TCRB chains is not to suggest that most TCRs are beta chain dominant. Many TCRs may exhibit alpha/beta chain codominance or even alpha chain dominance. Indeed, beta chain dominance could be a property of a minority of TCRs, but the majority of TCRs having convergent TCRB chains.

Finally, although it is not a focus of this study, the link between chronic antigen stimulation and autoimmunity suggests that TCR convergence may also have application to the identification of mechanistic or predictive biomarkers for autoimmune disease and infectious disease, particularly those involving chronic infection. To this end we note that elevated TCR convergence has been identified as a repertoire feature of salivary gland inflammatory lesions in individuals with Sjogren's syndrome (28), while convergent TCRs may be underrepresented in the peripheral blood of individuals with uncontrolled HIV infection (29).

## Data Availability Statement

All datasets generated for this study are included in the article/Supplementary Material.

## Ethics Statement

This study was approved by Roswell Park Cancer Institute (Buffalo, NY) internal review board review (IRB protocol # BDR 073116, Molecular techniques for the improvement of classification of pathology specimens) as per institutional policy for non-human subjects research, and UCSF IRB protocol CC#11858, Mechanism of pyrexia syndrome and immune response in patients, 11-07554. All human subjects gave written informed consent to participate in the protocol.

## Author Contributions

TL conceived the study, participated in its design and coordination, performed the statistical analysis, and drafted the manuscript. DT-H and GL carried out the immunoassays, participated in the sequence alignment, and helped draft the manuscript. JC carried out the immunoassays, curated sample response annotations, and edited the manuscript. CM procured samples and response annotations and edited the manuscript. DO participated in its design and helped draft the manuscript. LF conceived the study, participated in its design, and drafted the manuscript. LZ conceived of the study, participated in its design and coordination, performed the statistical analysis, and drafted the manuscript. All authors read and approved the final manuscript.

### Conflict of Interest

TL, DT-H, and GL are current employees of Thermo Fisher Scientific. JC and CM own stock in OmniSeq Inc. The remaining authors declare that the research was conducted in the absence of any commercial or financial relationships that could be construed as a potential conflict of interest.

## Supplementary Material

Supplemental Figure 1 **(A)** Clonality values for Oncomine and ImmunoSeq (*N* = 666) data sets. ImmunoSeq data corresponds to samples presented in Emerson et al. (12). Set 1 corresponds to eight samples used as part of the Sequenta-Oncomine cross-platform comparison, while Set 2 corresponds to 22 peripheral blood samples used to evaluate the predictive value of TCR convergence and clonality. **(B)** Convergence values for Oncomine and ImmunoSeq datasets. Set 1 samples analyzed by the Sequenta assay have significantly higher convergence values than the same samples analyzed by the Oncomine TCRB-LR assay. **(C)** Correlation between clonality and convergence in ImmunoSeq (*N* = 666) and **(D)** oncomine (Sets 1 and 2 combined, *N* = 30) datasets.

Supplemental Figure 2 Modeling ImmunoSeq convergence values as a product of substitution sequencing errors. A model was generated taking into account the sequencing depth per clone, and the likelihood that a substitution sequencing error would give rise to a synonymous mutation within the CDR3 of each clone. Observed vs. predicted values are presented for three hypothetical residual substitution sequencing error rates. Model fit was assessed by Spearman correlation. A residual substitution error rate of 8.5E-3 gave rise to convergence values that most closely fit the observed convergence values in the Emerson et al. (12) dataset.

Supplemental Figure 3 Clones detected following sequencing of 10E3, 10E4, and 10E5 sorted CD3 positive peripheral blood T cells from a healthy donor. Sorted cells were cultured in CTS™ OptiMem for 4 days followed by extraction of total RNA. The entirety of the RNA was used for library preparation via the Oncomine TCRB-LR assay followed by sequencing to saturation.

Supplemental Table 1 (Sheet 1) Key repertoire metrics for each sample presented in Table 1. Repertoire metrics were produced as a standard output of the Ion Reporter analysis pipeline. A detailed description of the metrics presented in this file is found within the glossary section of the user guide for the Oncomine TCRB-LR assay. (Sheet 2) Key repertoire metrics for samples evaluated as part of the cross-platform comparison.

Supplemental Table 2 Productive rearrangements reported following sequencing of 30 plasmid pool containing productive TCRB rearrangements presented in Sandberg et al. (10) and used to validate the BIOMED-2 primer set. Plasmids were pooled at equimolar input, followed by survey or deep level sequencing via the Oncomine and ImmunoSeq assays. ImmunoSeq assay was run by a contract research organization.

Supplemental Table 3 Limit of detection analysis of Oncomine TCRB and Archer Immunoverse HS TCR beta assays. **(A)** Average number of clones detected across PCR replicate libraries for Oncomine TCRB-LR and Archer Immunoverse HS TCR beta assays. Jurkat total RNA was spiked into total RNA derived from healthy donor PBL at 10E-5 or 10E-6 frequency, then libraries prepared from 25 to 300 ng RNA using the Oncomine TCRB-LR or Archer Immunoverse HS TCR beta assay. Two PCR replicates were performed for each input amount and spike in frequency. Libraries were analyzed using an equivalent depth (~2 M reads per library; libraries not sequenced to saturation) across the assays. **(B)** Fraction of libraries having detected Jurkat spike-in clone. **(C)** Average convergent TCR frequency across library replicates for Oncomine TCRB-LR and Immunoverse HS TCR beta assays. **(D–F)** Average number of clones detected, fraction of libraries having detected Jurkat clone, and average convergent TCR frequency for Oncomine TCRB-SR and Immunoverse HS TCR beta assays. Libraries were sequenced to saturation (>20 M reads each) via the Ion Torrent or lllumina platform. All experiments were performed by a contract research organization according to assay manufacturer specification.

Supplemental Data Sheets 1–7 Clonotyping data and repertoire metrics deriving from the Oncomine TCRB-LR assay and Ion Reporter analysis.
