# Supplementary material for: TCR Convergence in Individuals Treated With Immune Checkpoint Inhibition for Cancer
Source: Front Immunol. 2020 Jan 9;10:2985. doi: 10.3389/fimmu.2019.02985 (PMC6962348; doi:10.3389/fimmu.2019.02985)

Supplemental Figure 1A

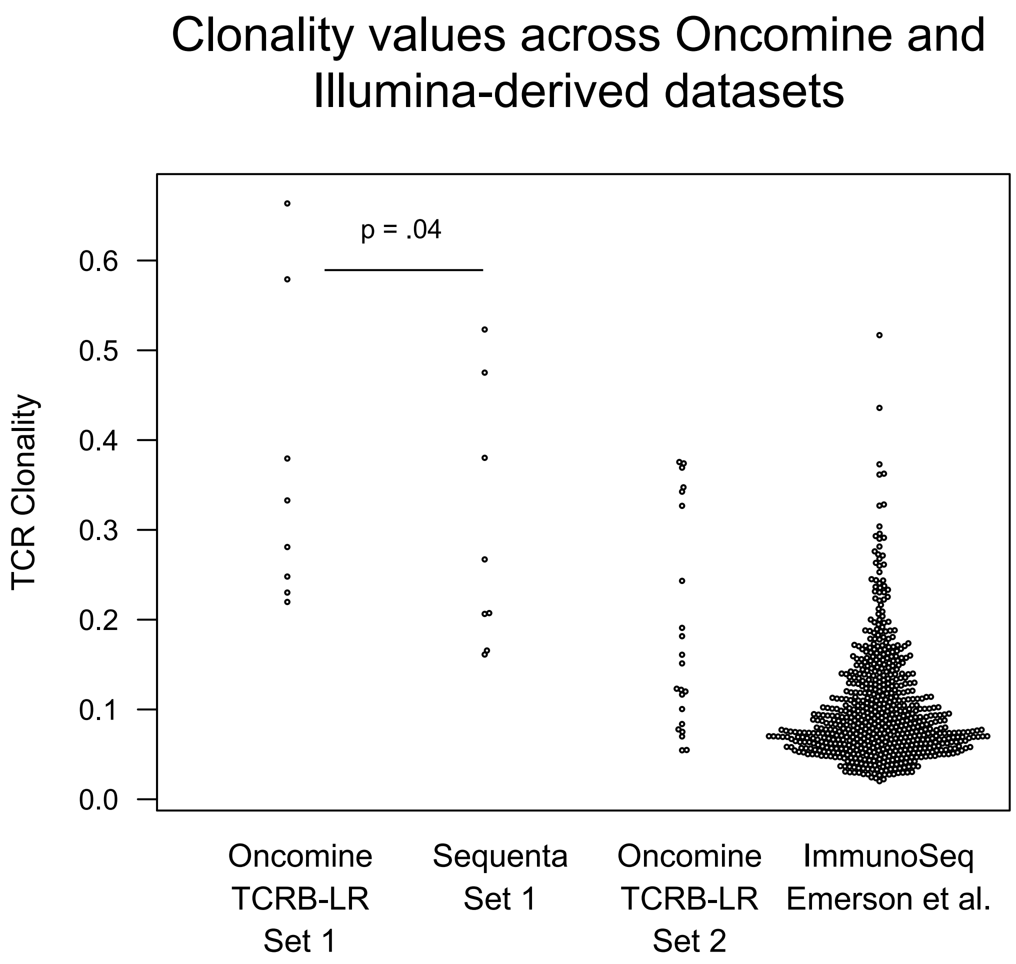

Supplemental Figure 1B

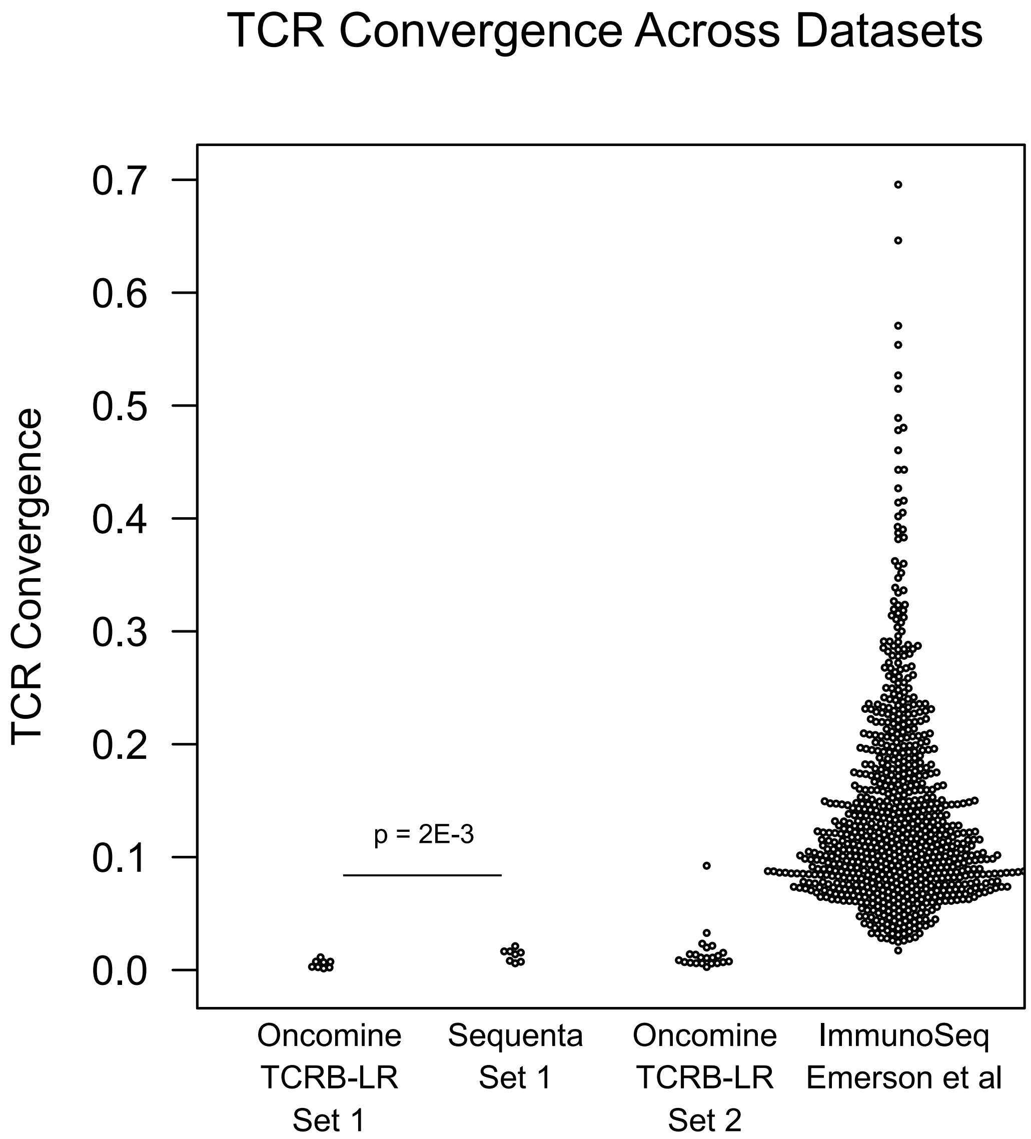

Supplemental Figure 1C

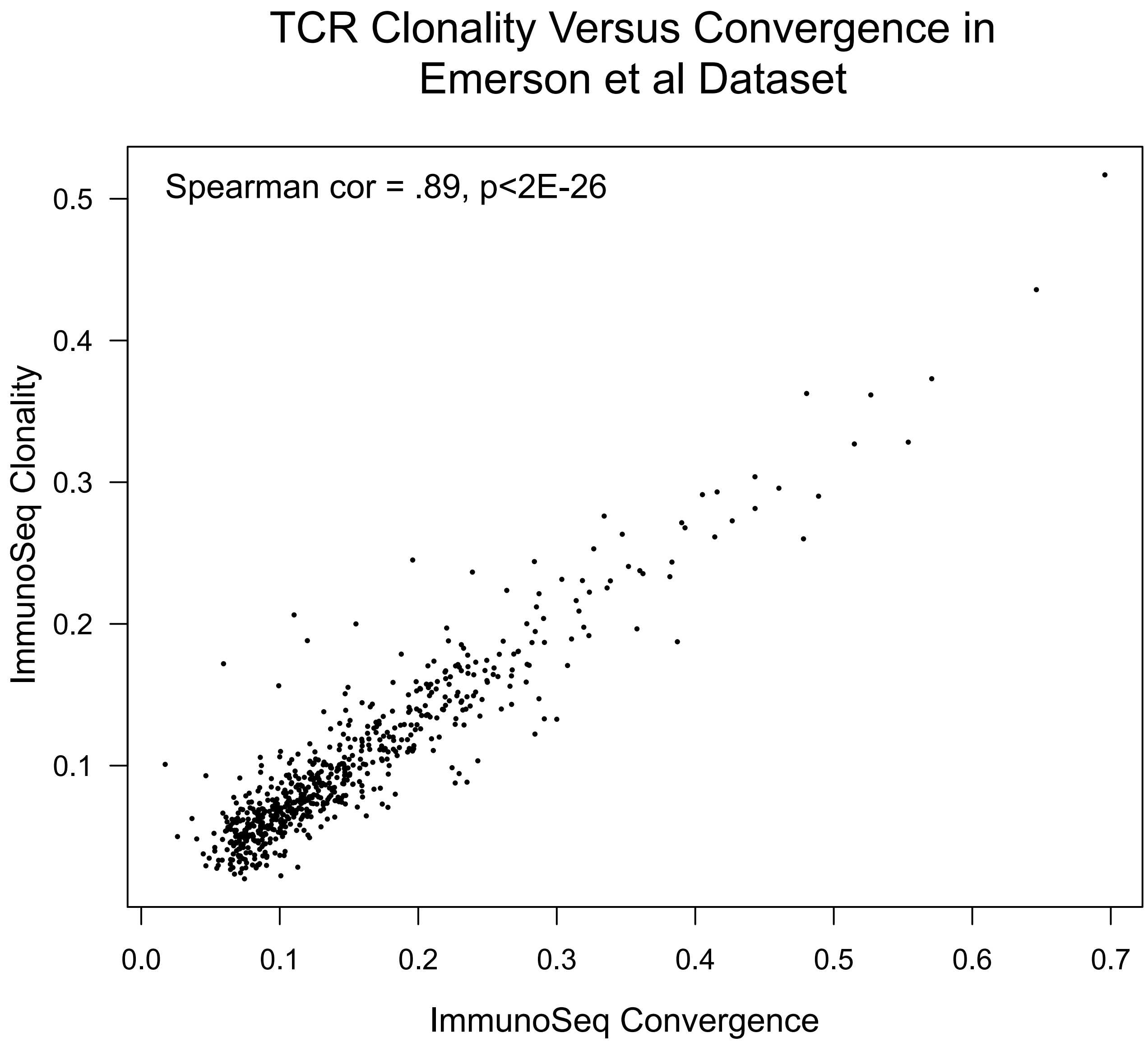

Supplemental Figure 1D

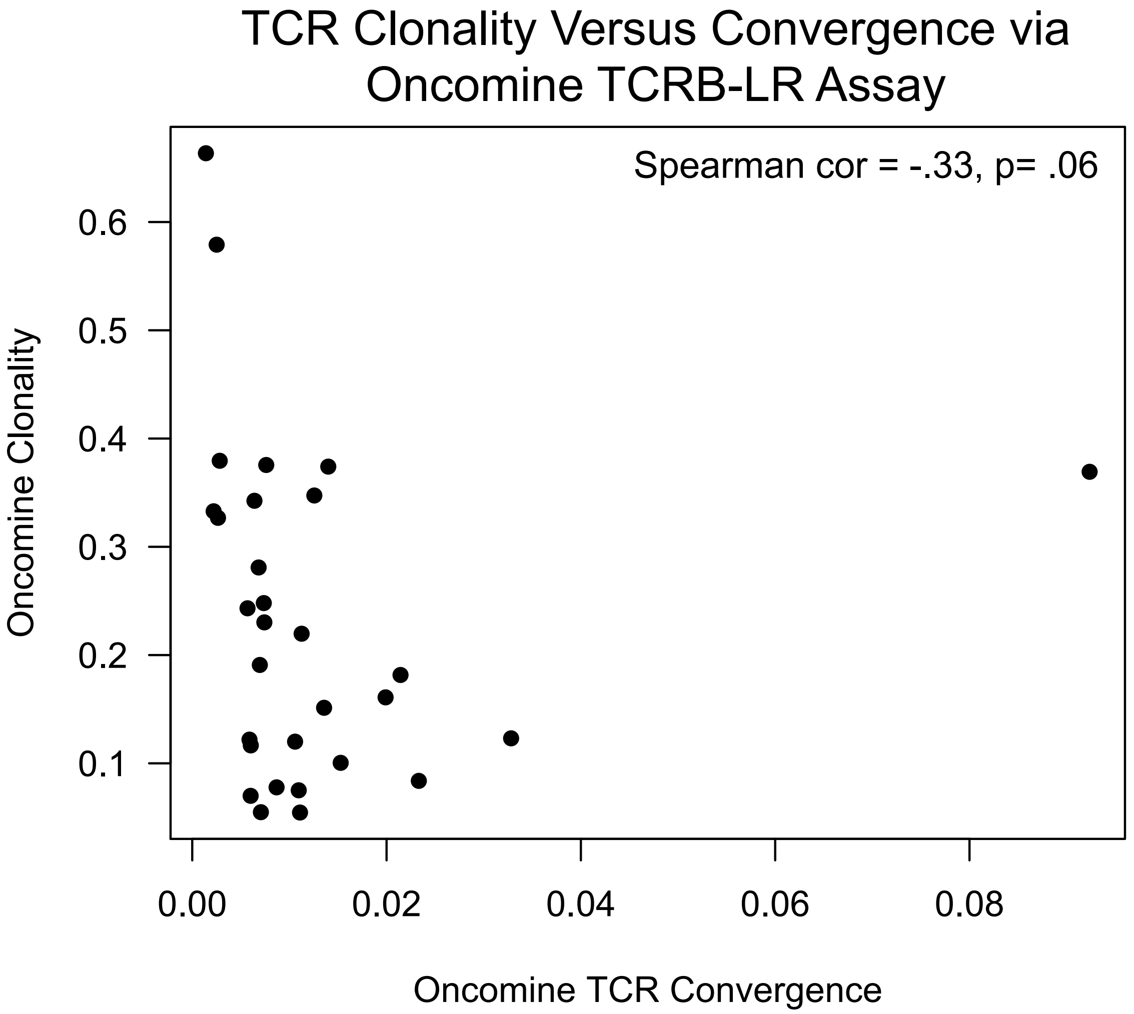

Supplemental Figure 2

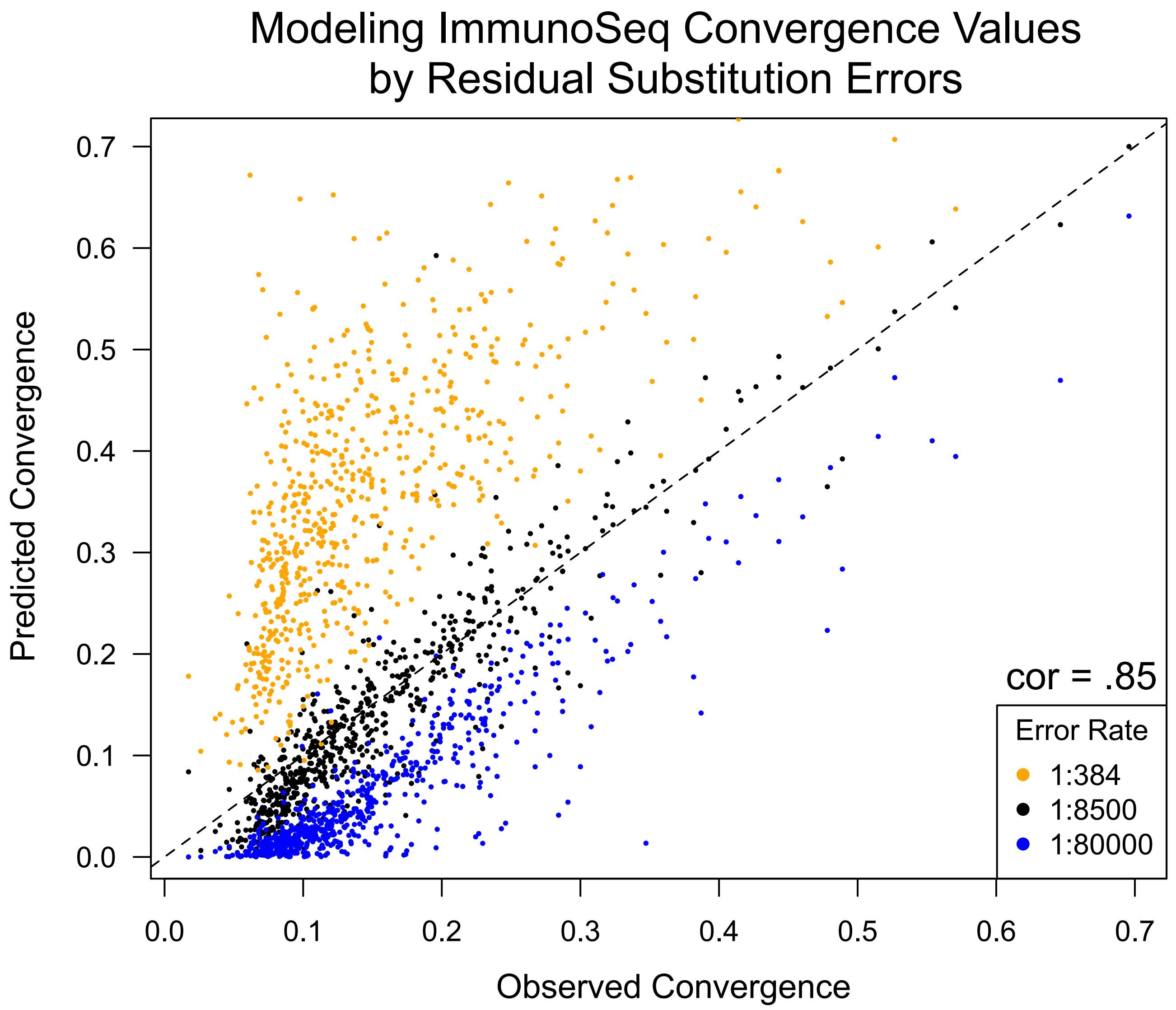

Supplemental Table 2

	Survey	Deep
Oncomine TCRB-LR	30	31
ImmunoSeq TCRB	55	84

Survey: 2 PCR replicates sequenced to ~1M reads depth
Deep: 6 PCR replicates sequenced to ~1M reads depth

Supplemental Figure 3

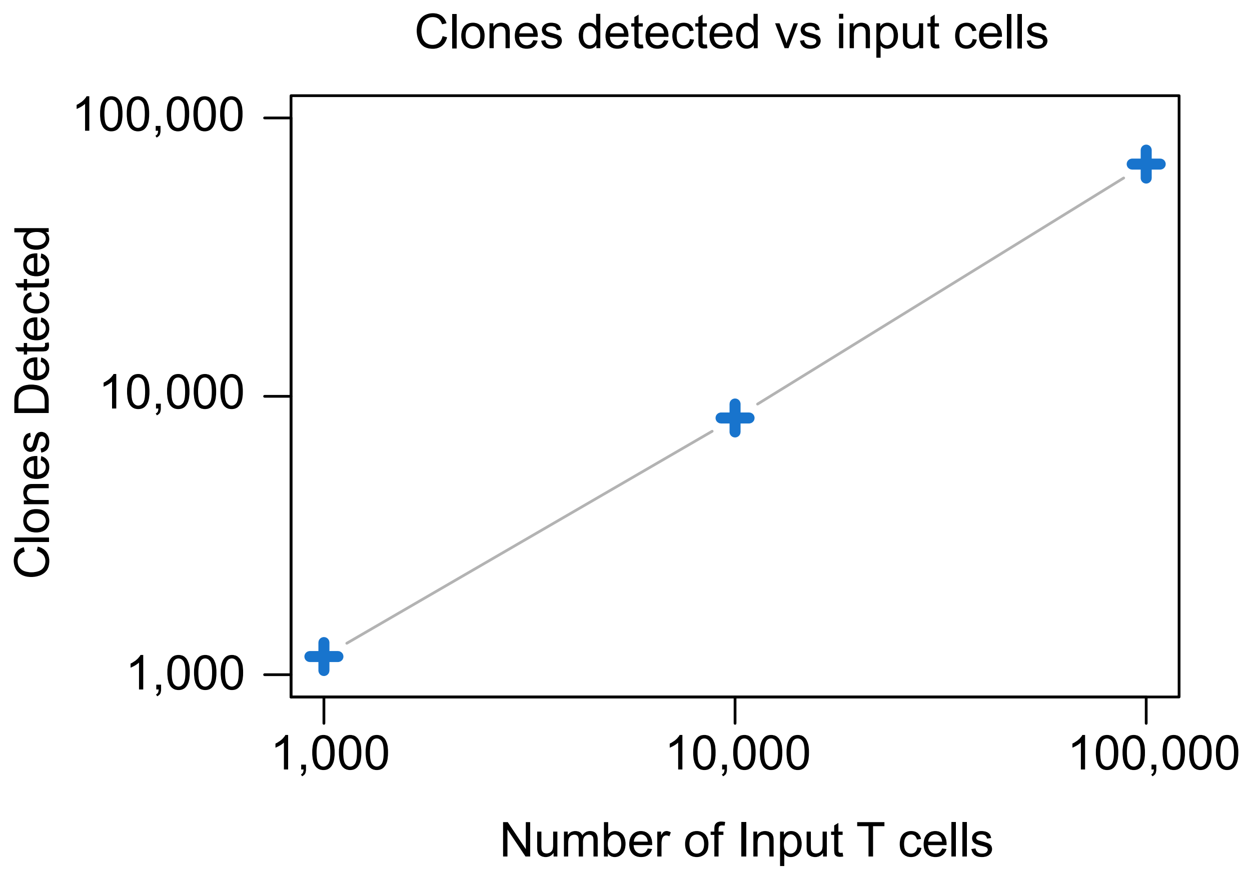

Supplement: Supplemental Figure 1 — (A) Clonality values for Oncomine and ImmunoSeq (N = 666) data sets. ImmunoSeq data corresponds to samples presented in Emerson et al. (12). Set 1 corresponds to eight samples used as part of the Sequenta-Oncomine cross-platform comparison, while Set 2 corresponds to 22 peripheral blood samples used to evaluate the predictive value of TCR convergence and clonality. (B) Convergence values for Oncomine and ImmunoSeq datasets. Set 1 samples analyzed by the Sequenta assay have significantly higher convergence values than the same samples analyzed by the Oncomine TCRB-LR assay. (C) Correlation between clonality and convergence in ImmunoSeq (N = 666) and (D) oncomine (Sets 1 and 2 combined, N = 30) datasets. [file Presentation_1.pdf]
